# Automatic Quantitative Analysis of Internal Quantum Efficiency Measurements of GaAs Solar Cells Using Deep Learning

**DOI:** 10.1002/advs.202407048

**Published:** 2024-12-04

**Authors:** Zubair Abdullah‐Vetter, Brendan Wright, Tien‐Chun Wu, Ali Shakiba, Ziv Hameiri

**Affiliations:** ^1^ University of New South Wales (UNSW) Sydney NSW 2052 Australia

**Keywords:** convolutional neural network, gallium arsenide, noise resilience, solar

## Abstract

A solar cell's internal quantum efficiency (IQE) measurement reveals critical information about the device's performance. This information can be obtained using a qualitative analysis of the shape of the curve, identifying and attributing current losses such as at the front and rear interfaces, and extracting key electrical and optical performance parameters. However, conventional methods to extract the performance parameters from IQE measurements are often time‐consuming and require manual fitting approaches. While several methodologies exist to extract those parameters from silicon solar cells, there is a lack of accessible approaches for non‐silicon cell technologies, like gallium arsenide cells, typically limiting the analysis to only the qualitative level. Therefore, this study proposes using a deep learning method to automatically predict multiple key parameters from IQE measurements of gallium arsenide cells. The proposed method is demonstrated to achieve a very high level of prediction accuracy across the entire range of parameter values and exhibits a high resilience for noisy measurements. By enhancing the quantitative analysis of IQE measurements, the method will unlock the full potential of quantum efficiency measurements as a powerful characterization tool for diverse solar cell technologies.

## Graphical abstract

The powerful application of deep learning is used in the analysis of internal quantum efficiency measurements from gallium arsenide solar cells. The novel approach not only automates the process with remarkable accuracy but also proves resilient against high levels of noise typically found in measurements.

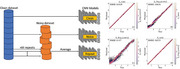

## 1 Introduction

The spectral response [SR(λ), where λ represents the wavelength] of solar cells is a key characterization technique that measures the output short‐circuit current (*I*_sc_) of a device under variable monochromatic light illumination.^[^ ^1^ ^]^ The spectral response can then be converted into the internal quantum efficiency (IQE), which is the ratio of the number of electrons the solar cell collects to the number of photons penetrating the device at each measured wavelength^[^ ^1^ ^]^:

(1) $$\;IQE\;\;\left(\lambda \right)=\frac{1}{1-R\left(\lambda \right)}\cdot \frac{hc}{q\lambda }\cdot SR\left(\lambda \right)$$

where *R* is the front surface reflection and h, c, and q are Planck's constant, the speed of light, and the elementary charge constant, respectively. Common uses of IQE measurements are to conduct loss analysis of solar cells,^[^ ^1^, ^2^ ^]^ calculate the expected *I*_sc_ of the device,^[^ ^3^ ^]^ and quantify key electrical and optical parameters of the solar cell.^[^ ^4^, ^5^, ^6^ ^]^ As a result, IQE measurements are a critical characterization technique for the research and development of solar cells.

There is a wide range of techniques to extract these electrical and optical parameters from IQE measurements. These techniques include the manual implementation of analytical equations to fit specific wavelength ranges of the measurement,^[^ ^5^, ^7^ ^]^ analytical equations designed to calculate the IQE along the entire spectrum,^[^ ^2^, ^4^ ^]^ or using modeling software to simulate the whole device and manually fine‐tune its parameters to achieve a good fit between the calculated and measured IQEs.^[^ ^6^, ^8^, ^9^, ^10^ ^]^ These manual analysis techniques pose several challenges such as a high sensitivity of the extracted parameters to the chosen wavelength ranges,^[^ ^5^, ^7^ ^]^ On the other hand, fitting the complete measurement typically requires manipulating many parameters.^[^ ^2^, ^4^ ^]^ In doing so, the user requires a deep knowledge of the studied device and an understanding of the correlations between the parameters.^[^ ^4^, ^9^, ^11^ ^]^ Additionally, the literature heavily focuses on silicon devices,^[^ ^2^, ^4^, ^12^, ^13^ ^]^ while only a limited number of approaches have been developed for the analysis of non‐silicon cells. In particular, gallium arsenide (GaAs) solar cells, where often only qualitative analysis has been provided from their IQE measurements.^[^ ^14^, ^15^ ^]^ Although only rarely discussed, an added challenge can arise in scenarios where multiple parameter combinations provide comparably good fits to the measurement.^[^ ^16^ ^]^ These cases result in a satisfactory fit for the measurement, but multiple sets of extracted parameters may be valid, and the most accurate solution cannot be easily distinguished.

One approach to automatically fit IQE measurements of GaAs solar cells involves a genetic algorithm,^[^ ^17^ ^]^ an adaptive search algorithm.^[^ ^18^ ^]^ Recently, the application of machine learning (ML) and deep learning (DL) has gained popularity for the automated extraction of material properties from device measurements.^[^ ^19^, ^20^, ^21^, ^22^, ^23^, ^24^ ^]^ These techniques have been employed in key areas: analyzing and extracting performance parameters from current‐voltage (I‐V) measurements,^[^ ^21^, ^25^ ^]^ processing luminescence images to identify defects and variations in solar cell properties,^[^ ^20^, ^26^ ^]^ and applying ML to in‐line measurements taken during various stages of solar cell production,^[^ ^27^, ^28^, ^29^ ^]^ Each approach involves training models to understand the measurement's behavior in relation to the relevant parameters. Therefore, the strength of ML and DL lies in their ability to learn complex relationships between material properties and measurement data. Such an application would be advantageous for the automated analysis of IQE measurements.

Therefore, this study introduces a DL method to automatically analyze and predict key performance parameters from IQE measurements of GaAs solar cells. This method eliminates the need to manually fit the IQE measurement or the time‐consuming task of manipulating many parameters in simulation software. Such an automated approach can significantly enhance the research and development of GaAs cells by unlocking the full potential of IQE measurements.

## 2 Methodology

### 2.1 Dataset

In this study, 40 000 cells (with an area of 0.25 cm^2^) were simulated in the open‐source Python package, SolCore.^[^ ^9^ ^]^ The default cell structure is an n‐type GaAs solar cell adapted from Tobin et al.^[^ ^30^ ^]^ From top to bottom, the cell consists of a front Al_0.79_Ga_0.21_As (aluminium:gallium, 79:21 ratio) window (18 nm), p‐type emitter (657 nm), n‐type base (3750 nm), and Al_0.3_Ga_0.7_As rear buffer layer (1000 nm).^[^ ^30^ ^]^ A dual‐layer ZnS/MgF_2_ (50; 100 nm) antireflection coating (ARC) was also applied in the simulation.^[^ ^30^ ^]^ Within each solar cell simulation, a random combination of the minority carrier diffusion lengths of the emitter (*L*_e_) and bulk (*L*_h_), and the surface recombination velocity of those regions (*S*_p_ and *S*_n_, respectively), was selected from a predetermined range of values. *S*_p_ and *S*_n_ were randomly sampled using a log scale to ensure a balanced dataset. The predetermined ranges (presented in **Table** **1**) were selected such that the extreme values of each parameter exhibit only minimal impact on the simulated IQE (hence, the IQE is almost insensitive to changes in these extreme values). The SolCore simulations were performed between 300 and 930 nm with 10 nm intervals.

**Table 1:** List of the simulated parameters and their range of values.

Parameter	Range
*L*_e_ [µm]	0.5–8
*L*_h_ [µm]	1–8
*S*_p_ [cm s^−1^]	1 × 10^3^–1 × 10^7^
*S*_n_ [cm s^−1^]	1 × 10^2^–1 × 10^6^

**Figure 1:**
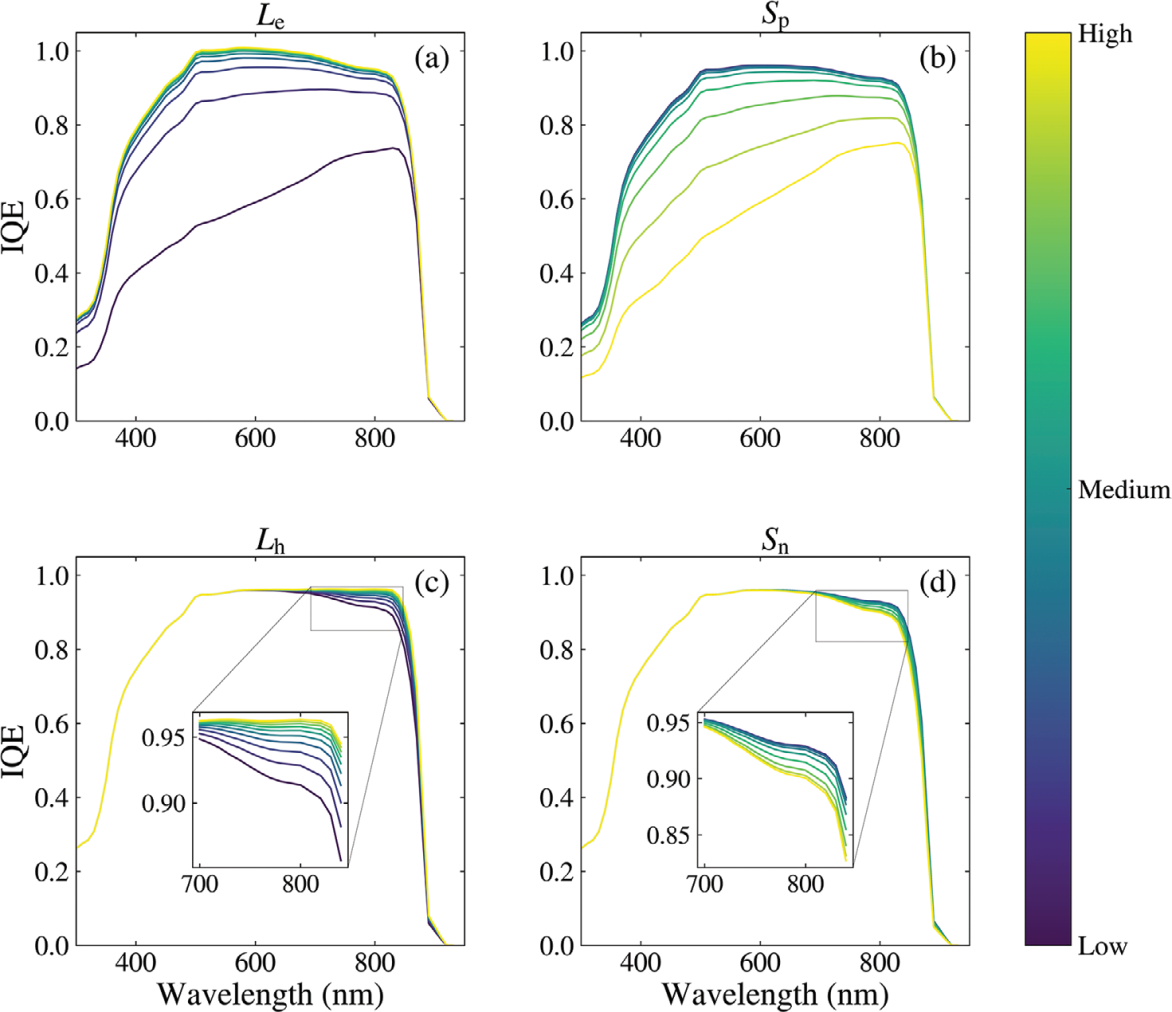
The effect of different parameters on the IQE: a) *L*_e_, b) *S*_p_, c) *L*_h_, and d) *S*_n_. The range of values was selected from Table 1.

The effect of each parameter on the IQE is presented in **Figure** **1**. As shown in Figure 1a,b, the emitter terms, *L*_e_ and *S*_p_, significantly affect the short wavelength region with a diminishing impact toward 900 nm. The IQE has a direct relationship with *L*_e_, such that it improves as *L*_e_ increases, whereas *S*_p_ has an inverse relationship, reducing the IQE at higher values. As evident in Figure 1a, when *L*_e_ increases to the maximum value (8 µm), its impact on the IQE decreases, showing a reduced sensitivity of the IQE to higher values of *L*_e_. This effect can also be seen in Figure 1b where the sensitivity of the IQE is also reduced as *S*_p_ decreases toward its minimum value (10^3^ cm s^−1^). As presented in Figure 1c,d, the bulk terms, *L*_h_ and *S*_n_, affect the longer wavelength range of 650 to 900 nm. Like the emitter terms, *L*_h_ has a direct relationship with the IQE while *S*_n_ has an inverse relationship. The reduced sensitivity of the IQE at the extreme values of the bulk parameters is also evident. However, unlike the emitter terms, the IQE is much less sensitive to the bulk parameters overall. Both the reduced sensitivity at the extreme values of each parameter and the overall bulk parameters (compared to the emitter parameters) are expected to cause issues for the DL predictions; these challenges are discussed in later sections.

### 2.2 Deep Learning Model

The proposed DL method utilizes a customized convolutional neural network (CNN)^[^ ^31^ ^]^ built using the Pytorch package.^[^ ^32^ ^]^ Deep learning methods have been successfully utilized in a wide range of applications due to their generalisability and accurate prediction performance.^[^ ^33^, ^34^ ^]^ Hence, their application in this ML task was expected to provide accurate prediction results. The custom CNN was constructed with two one‐dimensional (1‐D) convolution layers (with output channels of 8 and 16) and three fully connected linear layers. In the convolution layers, a kernel size of three was utilized, and the fully connected layers were ReLu (rectified linear unit) activated.^[^ ^31^ ^]^ The convolution layers allow the CNN model to extract more relevant information from the input data which is then utilized by the fully connected layers to predict the parameters.^[^ ^31^ ^]^ The model was trained up to 1500 epochs (training loops over the entire training set) with early stopping based on an adaptive learning rate function^[^ ^32^ ^]^ and an ADAM (adaptive moment estimation) optimizer,^[^ ^35^ ^]^ applied with a mean squared error loss function.

Before training, the dataset was split into a training set (80%) and a test set (20%). The training data was further split into smaller training and validation sets (also 80:20 ratio), where the validation set was used to ensure the model was not overfit to the training data.^[^ ^36^ ^]^ The training data was augmented with additional feature engineering steps, which were found to improve the CNN model further. Feature engineering is the extraction or transformation of the input data to enhance an ML model's prediction performance.^[^ ^36^ ^]^ The first feature engineering step was the addition of the IQE difference curve, which is the difference in the IQE values at each 10 nm interval of the measurement. The second feature engineering step was the application of principal component analysis (PCA), which is a popular dimensionality reduction algorithm that learns to transform datasets into shorter‐length feature vectors, aiding the interpretation of the original features.^[^ ^37^ ^]^ Beyond further improving the prediction performance, this step also reduced the training time of the model, lowering the number of epochs required by the CNN by three times.^[^ ^38^ ^]^ PCA of 20 components was applied separately to the IQE measurement and the IQE difference curve, resulting in 40 components that comprise the input feature vector. This approach trained a single CNN to predict all four parameters. Furthermore, if a different GaAs solar cell structure is produced, this procedure can be used to fine‐tune the CNN model using datasets generated utilizing the new structure.

To evaluate the trained models, the predicted and true values of the test set were compared. The final scores for each of the four parameters were determined using the root mean square error (RMSE) equation:^[^ ^36^ ^]^

(2) $$RMSE\;\;\left(y,\hat{y}\right)=\frac{1}{N}\;\sqrt{\sum_{i=1}^{N}{\left(y_{i}-{\hat{y}}_{i}\right)}^{2}}$$

where *N* is the number of samples, *y_i_* is the true value of the *i^th^* sample, and ${\hat{y}}_{i}$ is the corresponding predicted value. The predicted parameters were then input to SolCore to obtain the predicted IQE curves. Equation (2) was also used to evaluate the fit quality of the predicted IQE measurements, where an IQE RMSE ≤ 1.5 × 10^−3^ is classified as a good fit.

### 2.3 Noise Resilience

A robust approach must also be resilient to noise introduced by the measurement tool. Therefore, a 40 000 simulated GaAs solar cells dataset was augmented to incorporate noise commonly encountered in IQE measurements (a ‘noisy dataset’). Random Gaussian noise distributions with a mean of zero and a standard deviation of 0.005 were generated, representing noise found in typical IQE measurements. This magnitude of noise was chosen to be similar to the noise reported in Tobin's measurement of a similar GaAs solar cell design.^[^ ^30^, ^39^ ^]^ It represents a high level of noise that could be expected in IQE measurements. A different noise distribution was added to each simulated IQE curve. The same feature engineering steps described above (IQE difference and PCA) were applied before inputting the data into the CNN. The CNN model trained on the clean data was then tested on the noisy test set. The test set results of the models trained on both clean and noisy datasets are compared in Section 3.2. It is important to note that the PCA transform model was only trained on clean data, allowing the effect of the added noise to be transferred to the PCA components.

**Figure 2:**
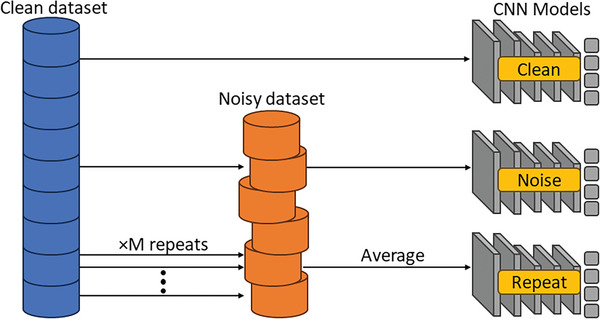
A dataset/ models flowchart. Three different CNN models were developed using the clean or the noisy datasets. The first CNN model was trained on clean IQE data, the second CNN model was trained on the noisy dataset, and the third CNN utilized *M* repeats and averaging of the noisy dataset.

It is common practice to repeat measurements multiple times and average them to obtain a better signal‐to‐noise ratio. Therefore, this procedure was investigated in the CNN pipeline. Each sample of the clean IQE dataset was randomly duplicated up to *M *= 25 times, and Gaussian noise distributions were randomly generated and applied to each duplicate. This represented collecting between one and 25 repeat measurements. The noisy duplicates were then averaged and the same feature engineering steps were applied. A new instance of the CNN model was also trained with this approach. To test this new CNN, separate test sets were produced by augmenting the clean dataset with *M* of 3, 9, 16, or 25, ensuring unique Gaussian noise was applied to each duplicate. This helped to identify an optimum number of repeats based on the prediction performance of each test set. In summary, two datasets (clean and noisy with added randomized Gaussian noise) were formulated for this study. Three different CNN models were developed using these datasets, as shown in the simplified flowchart in **Figure** **2**.

### 2.4 Validation

The CNN approaches were also validated using an IQE measurement of a GaAs solar cell from the literature. Using an online tool,^[^ ^40^ ^]^ an IQE measurement was extracted from a figure published by Tobin et al.^[^ ^39^ ^]^ The predicted four parameters by the CNN approach were fed into SolCore to generate the predicted IQE, which was then evaluated using Equation (2) to calculate the RMSE between the predicted and measured IQE. The IQE measurement was also manually fitted and the extracted parameters were compared to the CNN‐predicted values.

## 3 Results

### 3.1 Clean Dataset

**Figure 3:**
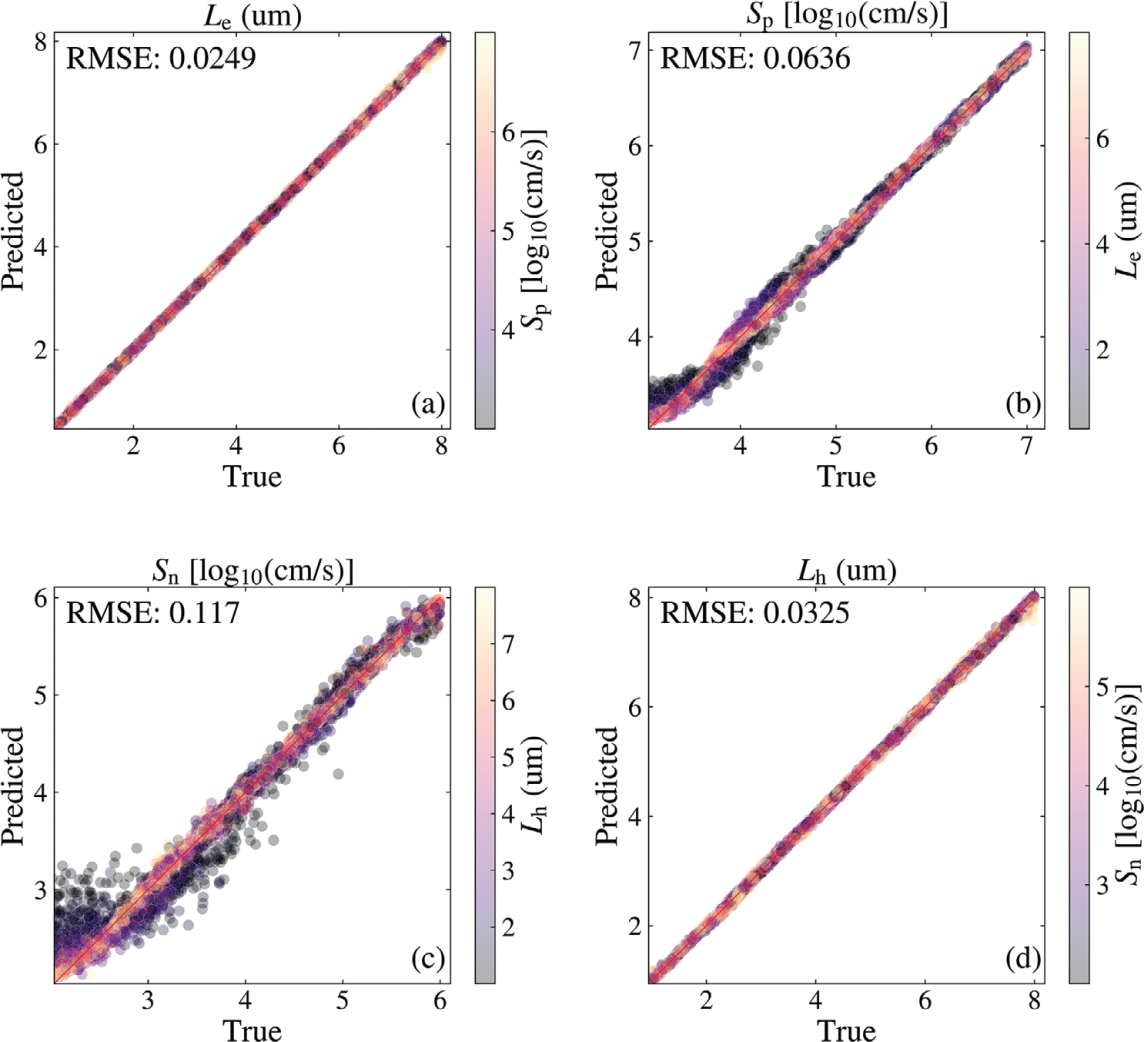
Predicted versus true plots of the predicted parameters: a) *L*_e_, b) *S*_p_, c) *S*_n_, and d) *L*_h_ of the CNN model. The color bars provide the values of the corresponding parameter within the same region of the cell.

The test set results of the CNN model are presented in **Figure** **3**. The color bars quantify the corresponding parameter within the same regions (i.e., emitter and bulk regions). As can be seen, the four parameters are predicted to have extremely low RMSEs, with most deviations from the true values attributed to a reduced sensitivity of the IQE to certain ranges of values. In Figure 3a, the few errors in *L*_e_ are in samples with the diffusion length reaching larger values (see Figure 1a), in combination with high values of *S*_p_ dominating the short wavelength region of the IQE. Both factors led to a reduced sensitivity of the IQE to the value of *L*_e_ and posed challenges for the accurate prediction of *L*_e_ in those few samples. Similarly, Figure 3b shows that the higher errors in the prediction of *S*_p_ are in samples where *L*_e_ dominates the short wavelength region combined with smaller values of *S*_p_ (see Figure 1b). Both factors reduce the sensitivity of the IQE to the value of *S*_p_, reducing the accuracy of the prediction. The reduced sensitivity of IQE to certain values of the bulk parameters was also the dominating factor contributing to the higher errors predominantly seen in Figure 3c and a few errors in Figure 3d.

Furthermore, another factor contributing to the higher errors in the bulk parameter predictions is the significant impact that the emitter terms have on the long wavelength region of IQE measurements (see Figure 1a,b). This effect is further described in Figure S1 (Supporting Information). As a large proportion of the higher errors (≥10% prediction error) are found in the parameter combinations with short diffusion lengths (see Figure 3b,c), this emphasizes the significant impact that the short *L*_e_ and *L*_h_ values (<1.5 µm) has on the IQE curves. Therefore, the CNN model would make more accurate predictions from IQE measurements of better‐performing GaAs solar cells. Nevertheless, despite those prediction errors, the CNN pipeline achieves impressively accurate predictions (≤10% error) across 95% of the test set.

**Figure 4:**
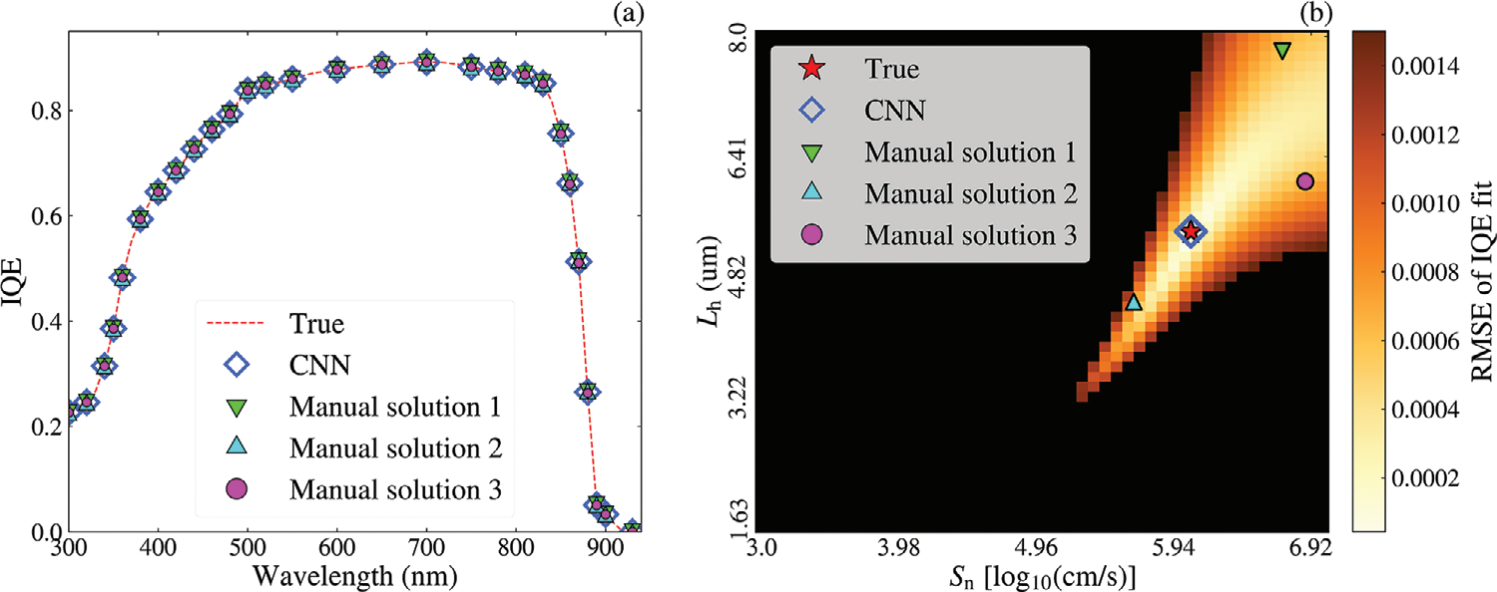
A representative example of multiple solutions providing a similar fit; a) the true IQE, CNN prediction, and manual fits. b) The RMSE heatmap, with the color bar set to a maximum of 1.5 × 10^−3^. The red star identifies the true values while the blue open diamond denotes the CNN model's prediction.

IQE RMSE heatmaps were generated for multiple samples to explore the test set results further. An interesting example is provided in **Figure** **4**. While great IQE fits were achieved, residual errors ≥10% in *L*_h_ were obtained. Figure 4a presents the predictions by the CNN model and three manual solutions. Despite significant errors in *L*_h_ (44%, 18%, and 12% in the manual fitting), the measured, predicted, and manually fitted IQEs are almost identical, scoring IQE RMSEs of 1.4 × 10^−4^ (CNN), 5 × 10^−4^, 4 × 10^−4^, and 6 × 10^−4^, respectively. The RMSE heatmap generated from the range of bulk parameters is shown in Figure 4b. The red star denotes the true pair of values, whereas the triangle, square, and circle show manually fit solutions. The blue open diamond represents the bulk parameters predicted by the CNN model, which is significantly more accurate than the manual solutions. The color bar is set to a maximum RMSE of 1.5 × 10^−3^, showing the vast range of bulk parameter combinations that result in a good IQE fit. As indicated in Figure 1 and discussed above, the significant impact of the emitter terms and the relatively small impact of the bulk parameters on the IQE is expected to cause reduced sensitivity of the IQE to the bulk parameters, which may lead to cases such as Figure 4. While these cases would cause issues with manual fitting, the CNN model achieved extremely accurate predictions, showing that the robust DL approach is ideal for handling such issues.

### 3.2 Noise Resilience

The results obtained by the CNN models, trained on either clean or noisy data, and tested on the **noisy** test set are provided in **Table** **2**. The clean test set results (Figure 3) are also included for easy comparison. When tested on the noisy data, the clean CNN model's prediction performance deteriorates and the RMSE scores increase by up to two orders of magnitude. Upon investigation of the higher error predictions, it is evident that the issue of decreased IQE sensitivity to specific parameter combinations became more pronounced with the added noise. This is especially evident in *L*_e_, with an RMSE score of 8.1. When trained on a noisy training set, it seems that the CNN model improves in distinguishing the IQE measurement from noise and overcomes the sensitivity issues encountered by the clean CNN. However, these results still suggest that the CNN is quite sensitive to noise. One method to boost the prediction results is repeat measurements.

**Table 2:** Summary of the CNN RMSE scores.

Parameter	Trained and tested on clean data	Trained on clean, tested on noisy data	Trained and tested on noisy data
*L*_e_ (µm)	0.0249	8.1	1.2
*S*_p_ [log_10_(cm s^−1^)]	0.0636	1.54	0.382
*S*_n_ [log_10_(cm s^−1^)]	0.117	2.06	0.587
*L*_h_ (µm)	0.0325	3.45	0.768

**Figure 5:**
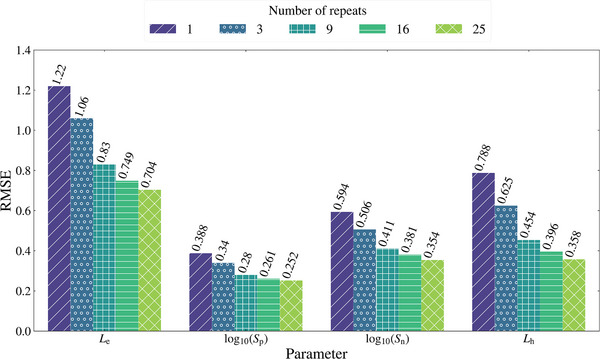
Results of the CNN model trained and tested on multiple repeat measurements and averaged.

The results of the CNN model trained to leverage the routine of repeat measurements are presented in **Figure** **5**. There is a clear improvement in the prediction performance of the CNN model as *M* increases. The CNN also obtains a small but negligible increase in RMSE for *M* = 1 (i.e., no repeats) compared to when it was trained on just one noisy IQE (Table 2; “Trained and tested on noisy data”). The lower RMSE in *L*_e_ indicates that this CNN is now more generalizable to the different levels of noise that could be found in IQE measurements. This is due to the deeper neural networks that were trained, where the training data was passed in batches (264 samples) to the model over many epochs.^[^ ^32^ ^]^ For each batch, a new *M* value is randomly chosen and applied to each sample in the data, allowing many cases to be passed to the model over the hundreds of epochs. Therefore, the new CNN overcomes the issues encountered by the previous CNN models and can better leverage the routine of repeat measurements.

**Figure 6:**
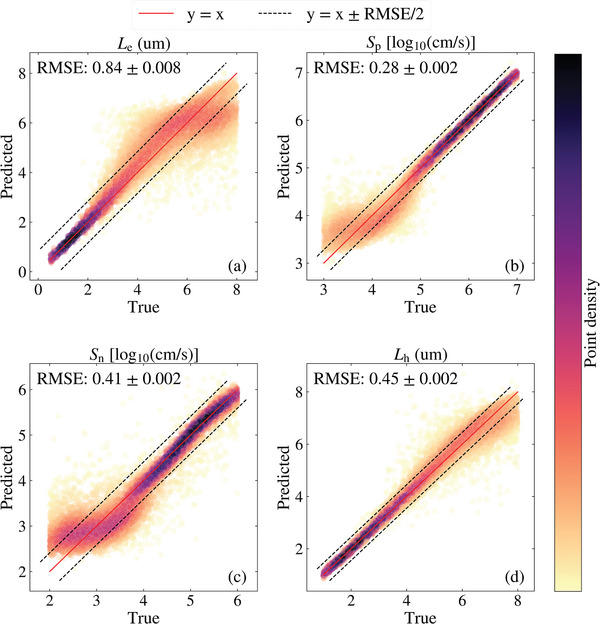
Predicted versus true plots of the test set from the CNN model trained to predict the four parameters with nine repeats of noisy IQE measurements (*M* = 9); a) *L*_e_, b) *S*_p_, c) *S*_n_, and d) *L*_h_. The RMSE scores represent the variation in prediction performance resulting from the random generation of the Gaussian noise. The color bar indicates the density of scatter points.

The prediction results of the CNN model tested on the *M* =  9 test set are presented in **Figure** **6**. This case was tested five times, and new noise distributions were generated for each test. The resulting RMSE scores show the small impact of randomly generated noise on the prediction. As expected, the areas of higher prediction errors corresponded to parameter combinations where the IQE is less sensitive. The color bar represents the density of the predicted values, which shows that while a small proportion of predictions exhibit high errors, most of the predictions align with the y = x line (≈77% are within the y = x ± RMSE/2 region). Hence, the routine of multiple repeat measurements allows the CNN model to achieve enhanced RMSE scores as the improved signal‐to‐noise ratio improves the data quality. While these enhanced results are an improvement from the noisy *M* = 1 case, they still represent diminished results from the clean test set. This indicates the inherent data limitations of the single IQE curve when such a magnitude of noise is in the measurement. Future studies may utilize more measurement data of these specific cases to obtain more accurate predictions. The predictions may also improve with further model optimizations using different architectures and hyperparameters. One such approach is discussed in Figure S2 (Supporting Information), where two‐dimensional (2‐D) CNN models were trained on 2‐D arrays of repeated IQE measurements. Nevertheless, these improvements may be only marginal due to the data limitations discussed.

### 3.3 Validation

The various ML models were validated using an IQE measurement of a GaAs solar cell extracted from the literature.^[^ ^39^ ^]^ A manual fitting attempt was conducted in SolCore, which involved testing numerous combinations of the four parameters. With extensive effort, an IQE RMSE of 0.0126 was achieved.

**Figure 7:**
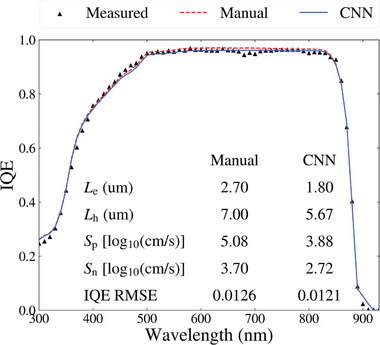
The prediction results of the CNN model trained on noisy IQE data compared to the manual fit of an IQE measurement of a GaAs solar cell extracted from the literature.^[^ ^39^ ^]^

**Figure 8:**
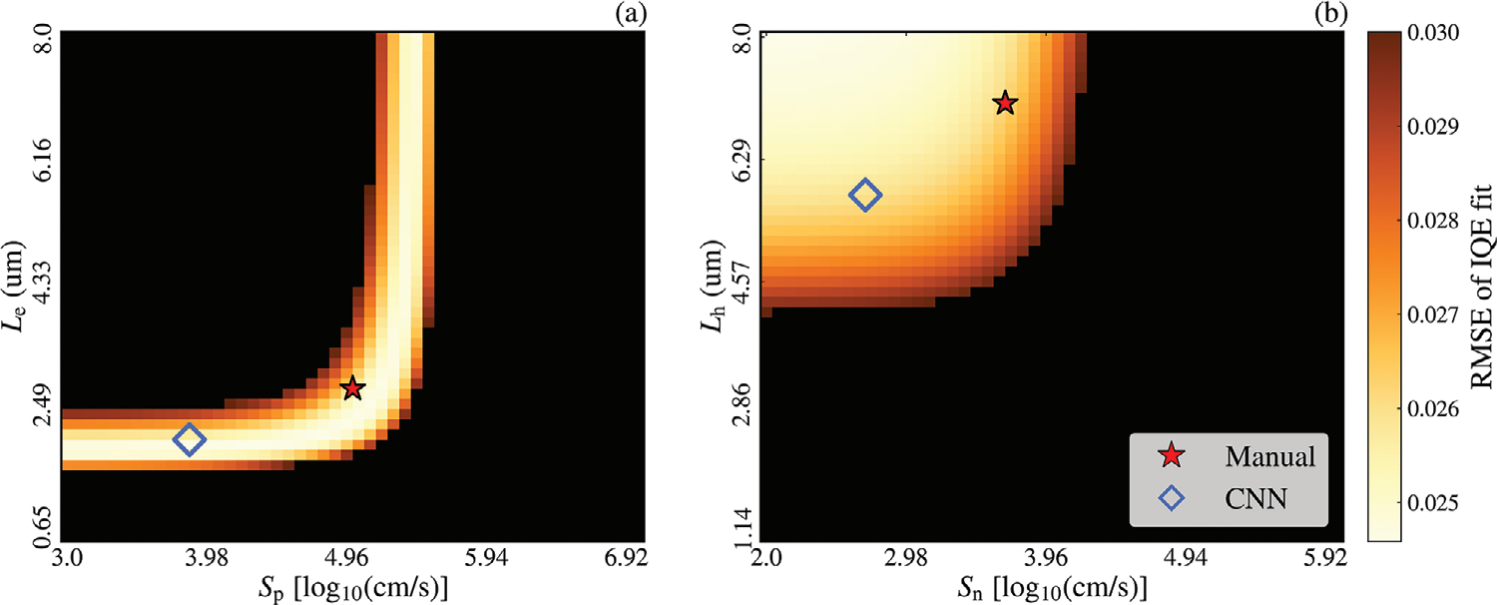
IQE RMSE heatmaps of the emitter and bulk parameter solution space for the validation data. The red star represents the manual fit values while the open blue diamond represents the CNN model's predictions.

The validation data was then tested on the trained DL models. The manual fitting and the results of the CNN model trained on the noisy IQE data are presented in **Figure** **7**. The manual fit and CNN‐predicted IQE curves closely match the IQE measurement, with deviations in the fits due to noise in the measurement. A pair of RMSE heatmaps representing the solution space for the emitter and bulk parameters are presented in **Figure** **8** where the manual and CNN solutions from Figure 7 are also provided. From the heatmaps, a wide range of solutions would provide great fits to the measurement, and this challenge is also exacerbated due to the noise in the measurement. For the emitter parameters, the CNN and manual fits achieve similar RMSE scores, although with a 24% difference in the value of log_10_(*S*_p_). For the bulk parameters, similar RMSE scores are achieved by both approaches, even though they have significant differences (≈20%) in magnitude. Figures 7 and 8 show that obtaining an accurate solution in IQE measurements with such a high noise level is complex. Regardless, the excellent fit between the CNN prediction and the IQE measurement demonstrates the capability of this approach for the fast, accurate, and automated analysis of IQE measurements of GaAs solar cells.

## 4 Conclusion

This study proposed the use of a CNN model to predict four key parameters from IQE measurements of GaAs solar cells. The CNN utilized two convolutional layers to extract relevant information from the IQE measurements automatically. The method obtained extremely accurate predictions from clean IQE measurements. When tested on IQE measurements with noise, the prediction performance declined due to the poor sensitivity to noise, which was compounded by the reduced sensitivity of the IQE to certain combinations of the four parameters. It was shown that training the models with noise in the training data improved the performance of the CNN model. Furthermore, the CNN model improves when utilizing a routine of repeat measurements. The trained models were also validated using IQE data extracted from the literature and IQE RMSE heatmaps indicated an extensive range of acceptable solutions. Regardless, the CNN model trained on noise outperformed the manual fitting on the validation data, obtaining a smaller RMSE and a faster prediction time. The proposed DL approach offers a fast, accurate, and automated analysis of IQE measurements of GaAs solar cells with possible extensions to other solar cell structures and technologies via fine‐tuning with the same training procedures. This approach unlocks the full potential of IQE measurements for the research and development of solar cell devices.

## Conflict of Interest

The authors declare no conflict of interest.

## Author Contributions

All authors contributed to the development of the methodology. Z.A.V. developed the models, did the analysis, and wrote the manuscript. Z.H. initiated and supervised the work.

## Supporting information



Supporting Information

## Data Availability

The data that support the findings of this study are available from the corresponding author upon reasonable request.
