# Supplementary material for: Automatic Quantitative Analysis of Internal Quantum Efficiency Measurements of GaAs Solar Cells Using Deep Learning
Source: Adv Sci (Weinh). 2024 Dec 4;12(4):2407048. doi: 10.1002/advs.202407048 (PMC11775520; doi:10.1002/advs.202407048)
Supplement: Supplementary file 1 — Supporting Information [file ADVS-12-2407048-s001.docx]

## **Supporting information**

#### **Effect of emitter terms on the bulk prediction**

The impact of the emitter terms on the long wavelength region of the IQE measurement [see Figure 1(a)] was expected to cause higher errors in the CNN model’s bulk parameter predictions. This effect was most prominent in samples with lower *L*_e_ values, which has the most significant impact on the long wavelength region of the IQE. This effect is evident in Figure S1, where the same prediction versus true plots of the bulk parameters from Figures 3(c) and 3(d) are presented; however, the colour bars now represent the values of *L*_e_. As expected, the higher errors are in samples where *L*_e_ is low. This effect, combined with those observed in Figures 3(c) and 3(d), results in the IQE becoming less sensitive to both *L*_h_ and *S*_n_.


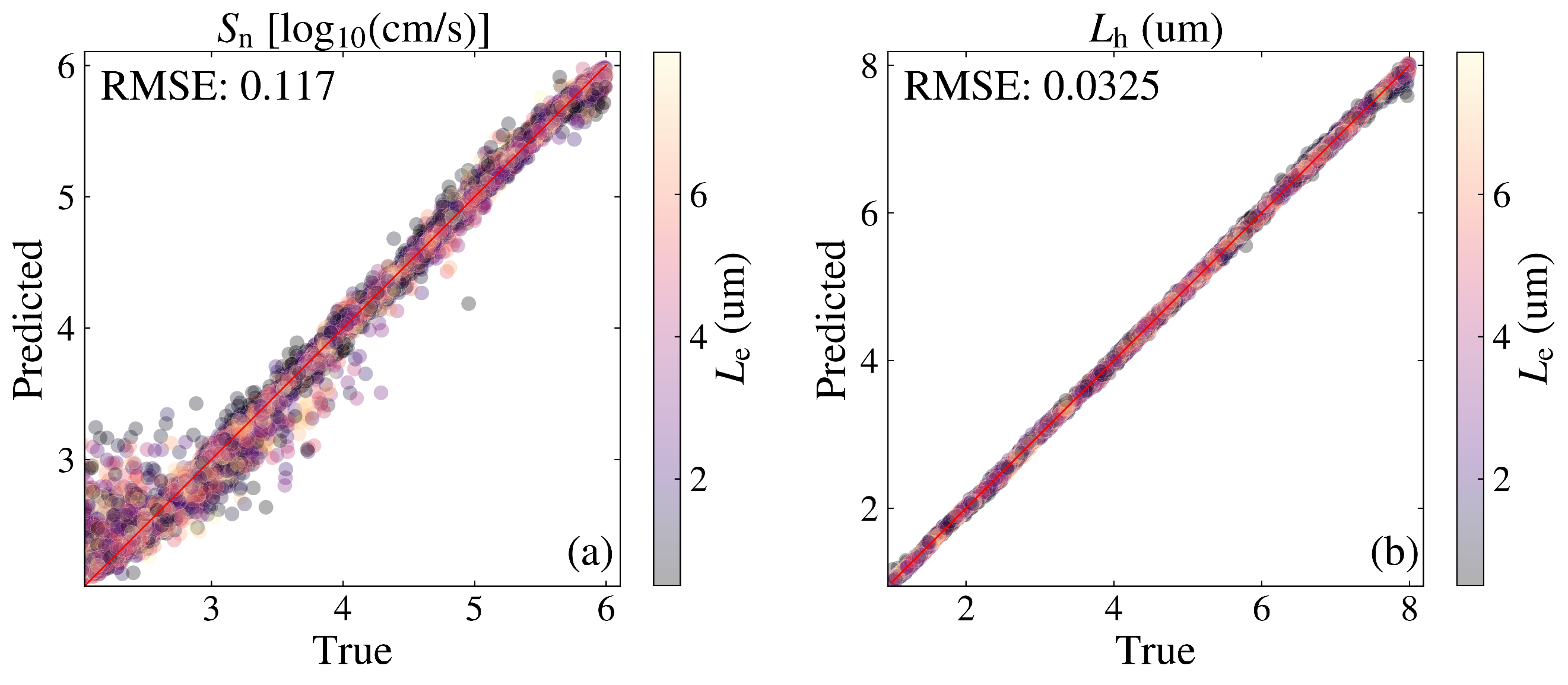


Figure S1 - Predicted vs. true plots of a) S_n_ and b) L_h_. The colour bars provide the values of L_e_.

#### **Two-dimensional CNN**

A two-dimensional (2-D) CNN approach was designed to utilise the common routine of multiple repeat measurements. Unlike the earlier models trained on the average of repeated measurements (reduced noise), the 2-D CNN models aimed to perform predictions on multiple noisy IQE measurements with the same magnitude of noise seen in the *M* = 1 case (no repeats). These CNN models were constructed with 2-D convolution layers, each with a kernel length of three and varying numbers of rows (*M* = 3, 9, 16, and 25). The datasets were augmented to include duplicate IQE measurements, each with unique noise distributions applied. These duplicated IQE measurements were stacked and input into the CNN models as 2-D arrays. The RMSE scores achieved by these 2-D CNN models are presented in Figure S2. The results of the 1-D CNN trained on repeated and averaged IQE measurements (Figure 5) are also included for easy comparison. Like the previous 1-D CNN, there are improvements in the RMSE scores with larger numbers of repeat measurements. In this scenario, the results indicate that the 2-D CNN has learned to better distinguish the underlying signal from the maximum magnitude of noise (*M* = 1) in the IQE measurements. However, there are only slight improvements in the RMSE scores compared to the 1-D CNN. This suggests that the patterns learned by the 2-D CNN from the stack of noisy IQE measurements are equivalent to the average of those measurements (Figure 5).


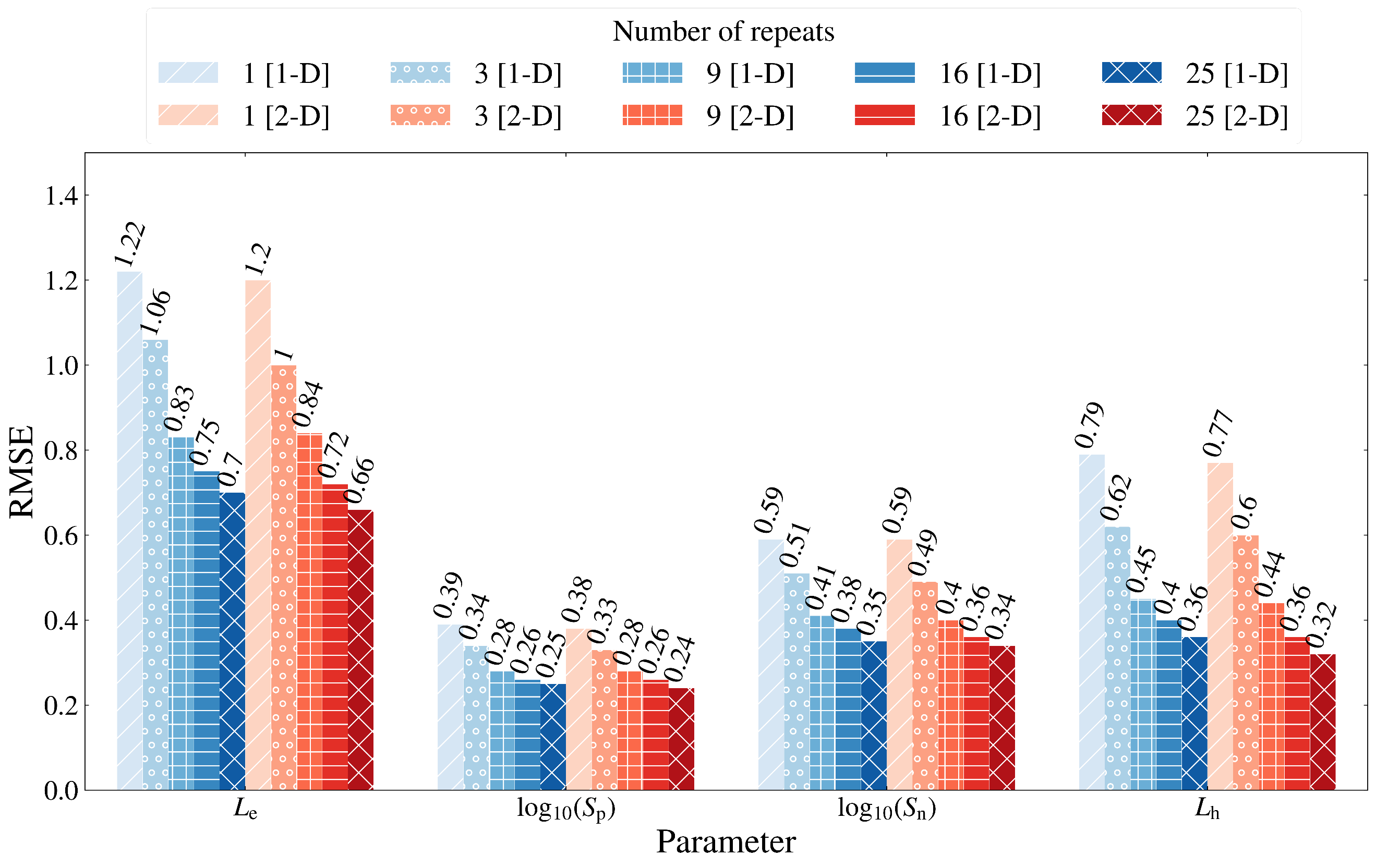


Figure S2 - Results of the 1-D and 2-D CNN models trained multiple repeat measurements. The repeat measurements were averaged before being input to the 1-D CNN, whereas the stack of repeat measurements was input to the 2-D CNN as a 2-D array. The models were tested on test sets with M = 1, 3, 9, 16 and 25.
